# Dermal Contributions to Human Interfollicular Epidermal Architecture and Self-Renewal

**DOI:** 10.3390/ijms161226078

**Published:** 2015-11-25

**Authors:** Kynan T. Lawlor, Pritinder Kaur

**Affiliations:** Hudson Institute of Medical Research, Clayton, VIC 3168, Australia; kynan.lawlor@hudson.org.au

**Keywords:** keratinocyte, epidermis, self-renewal, dermis, stem cell, microenvironment, vasculature, pericyte, endothelial, fibroblast

## Abstract

The human interfollicular epidermis is renewed throughout life by populations of proliferating basal keratinocytes. Though interfollicular keratinocyte stem cells have been identified, it is not known how self-renewal in this compartment is spatially organized. At the epidermal-dermal junction, keratinocytes sit atop a heterogeneous mix of dermal cells that may regulate keratinocyte self-renewal by influencing local tissue architecture and signalling microenvironments. Focusing on the rete ridges and complementary dermal papillae in human skin, we review the identity and organisation of abundant dermal cells types and present evidence for interactions between the dermal microenvironment and the interfollicular keratinocytes.

## 1. Introduction

Skin is a large, complex organ that is constantly renewed throughout life. The interfollicular epidermis is arranged in a spatial hierarchy with cells becoming more differentiated as they move to the outer layers and eventually shed. Differentiating cells are replenished by a basal layer of proliferating keratinocytes. Attempts to understand the complex dynamics that give rise to keratinocyte self-renewal have identified two main modes of renewal: a hierarchal model, whereby stem cells give rise to dividing transit, amplifying cells that produce differentiating daughters [1,2], and a single progenitor model [3,4,5]. Understanding the specific contribution of each type of renewal during homeostasis or challenge and how different conditions or tissue sites may favour a particular mode of renewal is the subject of ongoing investigation. Importantly, there is much to be learnt about how changes in the tissue microenvironment establish and modulate the organisation of keratinocyte populations to achieve sustained self-renewal.

Despite rapid advances in our understanding of skin stem cell biology, an interfollicular epidermal stem cell niche has yet to be unequivocally identified. The existence of such a niche is conceptually complicated by the nature of the tissue itself. The epidermis is large, with minimal cell movement, requiring a vast planar array of niche domains to produce a continuous, coordinated tissue. At the same time, regional cues must be integrated to modulate thickness and patterning at specific body sites. Whether a *bona fide* interfollicular epidermal stem cell niche exists or whether populations of keratinocytes intrinsically organise around neighbouring cells and the constraints of tissue architecture remains an open question.

While experiments in mice have contributed enormously to our fundamental knowledge of skin biology, some key features of human skin are not replicated in the mouse [6]. Human skin is thicker, with a lower density of hairs and a characteristic undulating pattern of rete ridges and alternating dermal papillae, which are largely absent in mice. Mouse skin has an underlying layer of muscle that is lacking in humans, such that the effects of mechanical stress and strain on the epidermis may be distinct in each case [6,7]. As our understanding of how cells respond to mechanical force increases [8,9,10,11], it will be important to consider how distinct tissue architecture and distribution of mechanical forces influences keratinocyte self-renewal [12,13,14].

**Figure 1:**
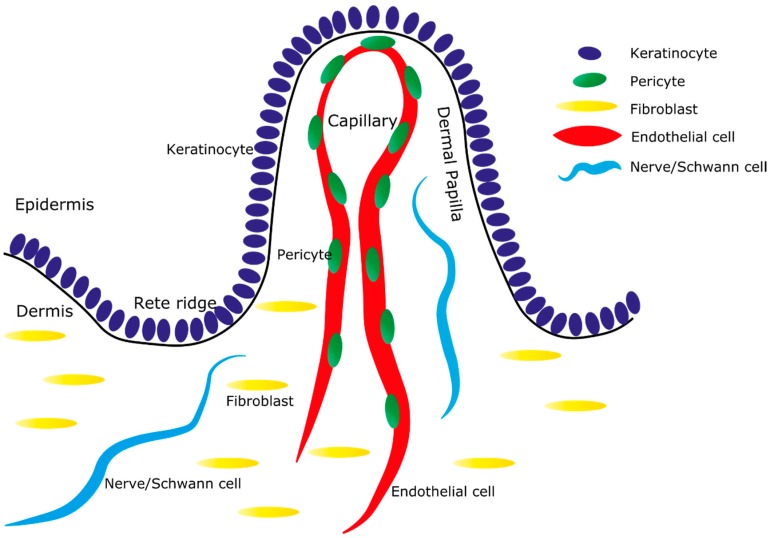
Architecture of the human epidermal-dermal junction. Simplified schematic representation of the human skin: The epidermis is made up of keratinocytes (dark blue, only the basal layer is shown) that sit atop a complex mix of dermal components. Capillary loops, containing endothelial cells (red) and pericytes (green), are located in the dermal papilla and extend up to the point where the dermis is closest to the external environment. Rete ridges occur where the epidermis is thickest and extend deep into the dermis. Fibroblasts (yellow) are present throughout the dermis. Nerves and Schwann cells (light blue) are present in a complex arrangement throughout the dermis. A single ridge and papilla are shown, though this pattern extends in a planar direction across the epidermis.

In this review, we focus on a particular feature of the human epidermal-dermal microarchitecture: the undulating pattern of rete ridges and dermal papillae within the interfollicular epidermis (Figure 1). We examine evidence that the properties of the keratinocytes, as well as the architecture of the dermis are organised around this patterned structure, providing a paradigm to examine how dermal heterogeneity supports epidermal homeostasis. We will also review the evidence for interactions between specific dermal cell types and the interfollicular epidermis. The important contributions made by the immune system to skin biology are outside the scope of this review, but have been thoroughly reviewed elsewhere [15,16,17,18,19].

## 2. Human Interfollicular Epidermal Architecture and Stem Cells

Evidence thus far suggests that the rete ridge pattern may correlate with the organisation of the self-renewing keratinocyte compartment; however, the precise location of distinct populations remains uncertain. Early labelling studies in primate plantar skin identified a bias in the position of labelled cycling cells toward the tips of the deep rete ridges [20]. In human skin, β-1 integrin expression is highest within the keratinocytes at the top of the dermal papillae in the “trough” that complements the rete ridges [21,22]. Marking the position of cycling cells, using Ki67 antigen or BrdU incorporation and early differentiating cells that express Keratin 10, revealed a spatial bias of these populations away from regions of high β-1 integrin expression [22]. Thus, a population of slow cycling cells, previously shown to express high levels of β-1 integrin and to possess the properties of stem cells [22,23], is likely located in regions of high beta-1 integrin expression at the tip of the dermal papillae. These findings suggest that keratinocyte renewal may involve a complex movement of cells laterally and down the rete ridge, as well as along the basal-apical axis to maintain tissue architecture. Interestingly, the sites of high β-1 expression appear to be body site specific: at the tip of the dermal papillae in breast, foreskin and scalp, but at the tip of the deep rete ridge in palm and foot skin [21,22]. The basis for this is unknown, but suggests that keratinocyte self-renewal may be organised around local differences in tissue architecture that are yet to be determined.

Work from our laboratory has identified quiescent epidermal stem cells within the interfollicular epidermis, defined by high α-6 integrin expression and low CD71 expression [24,25,26]. In adult skin, the α-6 bright stem and transit amplifying populations express keratin 15 (K15), while staining reveals the spatial restriction of K15 expression to the tips of the rete ridges [27]. Thus, the stem cell compartment in adult skin appears to be located at the tip of the rete ridges [27]. In neonatal foreskin tissue, K15 is expressed uniformly throughout the basal layer, suggesting that either the correlation between K15 expression and the stem/transit amplifying cells changes or that the spatial restriction of the stem cell compartment may vary with age or tissue site.

Constrained by the limitations of working with human tissue, there are many important questions regarding the organisation of the human interfollicular epidermal stem cells that remain unanswered. Lineage tracing experiments, to investigate whether a single progenitor mode of renewal occurs in human skin, are not possible. Nonetheless, understanding how the constraints from structural features, such as the rete ridges, interact with different models of self-renewal will be an important area of future study. As more precise ways to define tissue architecture or dermal cell populations are developed, it may be possible to identify extrinsic factors that account for the spatial organisation of keratinocyte self-renewal. 

## 3. Rete Ridges and Capillaries

To understand the role of the rete ridges in regulating the basal keratinocytes, it will be necessary to understand how the dermis and epidermis interact to give rise to this undulating structure.

The rete ridges increase the surface area of the epidermal-dermal junction, providing mechanical strength to the skin [28]. Indeed, ageing results in a flattening of these ridges, giving rise to skin that is weak and more likely to be damaged [29].

The rete ridge pattern also increases the surface area of the capillary-epidermal interface to improve nutrient supply to the avascular epidermis. In human skin, a repeating array of capillaries emanates from the superficial vascular plexus and loop up in close proximity to the epidermal-dermal interface [30]. The position of capillary loops is precisely aligned with the structure of the epidermis, such that capillaries are contained within the dermal papillae that occur between rete ridges (Figure 1).

The dermal capillaries supply the skin with nutrients and oxygen, though oxygen may also be absorbed from the external environment [31]. Skin has recently been shown to regulate the systemic response to hypoxia by sensing external oxygen levels through HIF1-α expression in keratinocytes [32]. Thus, the role of the dermal capillaries may extend beyond nutrient supply. The dermal vasculature plays an important role in temperature regulation by modulating blood flow to increase or decrease heat lost to the external environment [33,34]. Interestingly, this appears to be regulated largely by increasing the blood flow through anastomoses in the superficial plexus, rather than the dermal capillary loops [34].

Given the important functional basis for the organisation of the dermal vasculature, it is likely that cues from the vasculature help to establish the architecture of the epidermal-dermal interface. The pattern of dermal capillaries emerges early during embryogenesis coincident with the development of the epidermis [35,36,37]. By birth, the skin possesses a mature capillary architecture; however, little is known about the mechanisms by which the epidermis organises around the capillaries to achieve this state. Evidence from other organs suggests an important role for the developing vasculature in tissue morphogenesis [38], though it is presently unknown how similar influences may apply in the skin. The identity of vascular cues that influence skin morphogenesis may shed light on the manner in which keratinocytes are organised to maintain a structural relationship with the dermis.

## 4. Endothelial Cells and Pericytes

The capillary loops are made up of endothelial cells, forming junctions to create tubular vessels. Cultured dermal endothelial cells have been shown to support organotypic skin culture [39], and endothelial cells have been included in artificial skin substitutes as a means to improve engraftment [40]. In the latter case, endothelial cells do not organise to form a normally-patterned capillary network, and keratinocytes do not form rete ridge structures, suggesting that interactions that give rise to this spatial arrangement do not occur in this system.

The second major component of the capillary loops is the pericyte population. These cells ensheath the microvasculature forming the interface between the endothelial cells and the surrounding tissue environment [41]. Though pericytes have been extensively studied in other tissues [42], the specific properties and role of dermal pericytes in regulating the skin are only beginning to be understood. Pericytes are known to contribute to fibrosis [43], and pericyte coverage has been shown to decrease during aging [44], though the functional impact of this change is unknown. Our laboratory previously showed that pericytes isolated from primary tissue are able to improve keratinocyte regeneration in an organotypic culture model [45]. Given this functional role and the position of pericytes lining the capillary loops, we propose that pericytes may influence the basal keratinocytes by regulating the architecture or signalling environment at the epidermal-dermal junction. Studies are currently underway to further examine the role of pericytes in skin homeostasis. Pericyte populations isolated from multiple tissues, including human skin, have been shown to display the properties of mesenchymal stem cells [45,46]. Thus, the skin provides an exciting opportunity to examine the role of this population in regulating tissue homeostasis and regeneration.

The perivascular environment has been identified as a niche component in the hair follicle [47], haematopoietic [48,49], neural [50], adipocyte [51] and myofibre stem cell niches [52]. Though no evidence exists for such an interaction with the epidermis, the close structural association of the capillary network with the basal epidermis in human skin warrants further investigation into the interactions between these populations. Dissecting the contributions of the endothelial cells and pericytes as contributors to the skin microenvironment will be complex as the two cell types are physically associated and functionally dependent on one another.

## 5. Nerves

Human skin acts as the interface by which the body senses the physical environment. Recent work has shed light on the complex repertoire of touch sensors within the mammalian skin [53]. Spatially, this sensory system comprises a complex array of nerves and supporting Schwann cells that make up a significant population within the dermis [54,55]. Innervation appears to influence keratinocyte proliferation [56,57,58] and has been shown to induce specific stem cell markers in interfollicular keratinocytes [59]. It has been proposed that in some instances, the structure of the epidermis may be arranged in a way that optimizes the transduction of sensation to specific nerves [60]. Sensory Merkel cells are renewed by a niche of specialised touch dome keratinocytes that are located at the tip of the rete ridge in mouse foot pad [61], though the functional requirement for this positioning is not certain. As further studies characterise the interactions between neurons, Schwann cells and the epidermis, as well as other dermal components, such as the vasculature, it may be possible to incorporate these into a broader model of epidermal homeostasis. In particular, it will be important to understand how cells in the sensory epidermal structures interact with neighbouring interfollicular keratinocytes.

## 6. Fibroblasts

Fibroblasts are responsible for secreting matrix proteins and collagen that forms the bulk of the dermis and have long been recognised as essential for epidermal growth [62,63]. Heterogeneity within the dermal fibroblast population is being increasingly uncovered, providing important insights into the role of these cells in epidermal homeostasis [64,65]. Previous studies have shown that fibroblasts isolated from human papillary *vs.* reticular dermis possess distinct properties and abilities to promote keratinocyte growth [66,67,68,69,70]. Similarly, fibroblasts from different anatomical sites have distinct transcriptional profiles [71,72]. Recent studies in mouse have identified fibroblast sub-populations that are specified during development and contribute differentially to skin development and homeostasis and under conditions of challenge [73,74].

Fibroblasts from different sites also appear to have a differential ability to promote vascular tube formation *in vitro* [75], highlighting the complex cross-talk that exists within the dermis. Further studies, particularly using mouse lineage tracing, may shed led light on the important interactions between dermal populations during development and homeostasis. As the field gains a better understanding of fibroblast heterogeneity, it may be possible to identify subsets that make specific contributions to keratinocyte self-renewal.

## 7. Adipocytes

Though historically overlooked, recent studies highlight a role for the dermal adipocytes in skin homeostasis and wounding [76,77,78,79,80]. Few data are available for human skin; however, mouse developmental studies have identified a specific adipocyte population in the dermis arising from a progenitor population that also gives rise to fibroblasts [73,81]. Epidermal signalling appears to modulate the differentiation of precursors into adipocytes [78], and adipocytes influence the hair follicle niche [76].

Though the adipocytes are not in close proximity to the epidermis, factors secreted by the dermal adipocyte layer may be important modulators of the properties and identity of other dermal cell types. Adipocytes may also support the mechanical properties of skin [82], though this area remains largely unexplored. A close association exists between the development of adipocytes and the vasculature [83], and adipocytes precursors reside within the adipose vasculature [51]. Interestingly, pericytes reside within a similar vascular location and possess mesenchymal stem-like properties, including an ability to differentiate into an adipocyte lineage [45,46]. The specific reservoirs from which different dermal populations are renewed during homeostatic conditions or challenge will be an interesting area of future investigation.

## 8. Dermal Heterogeneity and the Basement Membrane

The basement membrane separating the epidermis from the dermis is composed of structural proteins that anchor the epidermis and provide signalling cues to the keratinocytes [84,85]. Loss of basement membrane components is associated with skin blistering diseases [86,87]. Basement membrane proteins bind specific integrins that define keratinocyte stem cell identity and prevent keratinocyte differentiation [21,22,23,24,25,26,84]. *In vitro* studies suggest an important role for dermal fibroblasts in generating a homeostatic basement membrane [88,89,90]. Similarly, increased laminin α-5 levels, associated with healthy skin [91], are seen when pericytes are included in organotypic culture [45]. The basement membrane is likely a critical determinant of how cues from the dermis influence the epidermis; however, the precise contributions of different human dermal cell populations to regional basement membrane composition *in vivo* are presently unknown.

## 9. Dermal Heterogeneity for Regenerative Medicine

In regenerative medicine, there is much interest in creating improved skin substitutes that more faithfully mimic natural skin [92,93]. Progress towards this goal has been limited by a lack of understanding of the complex topology and interactions within the dermis and at the epidermal-dermal junction. By understanding how different components define specific microenvironments within the skin, it may eventually be possible to recreate a functional niche to promote homeostatic interfollicular epidermal self-renewal. Recreating features, such as the rete ridge pattern, may impart skin with improved mechanical properties and allow for more efficient vascularisation. Similarly, a better understanding of the interactions between the epidermis and the nervous system may allow the design of artificial tissue that correctly incorporates nerves to restore sensory function.
